# KinderMiner Web: a simple web tool for ranking pairwise associations in biomedical applications

**DOI:** 10.12688/f1000research.25523.1

**Published:** 2020-07-30

**Authors:** Finn Kuusisto, Daniel Ng, John Steill, Ian Ross, Miron Livny, James Thomson, David Page, Ron Stewart

**Affiliations:** 1 Morgridge Institute for Research, Madison, WI, 53715, USA; 2 Computer Sciences Department, University of Wisconsin-Madison, Madison, WI, 53706, USA; 3 Department of Molecular and Cellular Biology, University of California, Santa Barbara, Santa Barbara, CA, 93117, USA; 4 School of Medicine and Public Health, University of Wisconsin-Madison, Madison, WI, 53706, USA; 5 Department of Biostatistics & Bioinformatics, Duke University, Durham, NC, 27710, USA

**Keywords:** Text mining, web application, KinderMiner

## Abstract

Many important scientific discoveries require lengthy experimental processes of trial and error and could benefit from intelligent prioritization based on deep domain understanding. While exponential growth in the scientific literature makes it difficult to keep current in even a single domain, that same rapid growth in literature also presents an opportunity for automated extraction of knowledge via text mining. We have developed a web application implementation of the KinderMiner algorithm for proposing ranked associations between a list of target terms and a key phrase. Any key phrase and target term list can be used for biomedical inquiry. We built the web application around a text index derived from PubMed. It is the first publicly available implementation of the algorithm, is fast and easy to use, and includes an interactive analysis tool. The KinderMiner web application is a public resource offering scientists a cohesive summary of what is currently known about a particular topic within the literature, and helping them to prioritize experiments around that topic. It performs comparably or better to similar state-of-the-art text mining tools, is more flexible, and can be applied to any biomedical topic of interest. It is also continually improving with quarterly updates to the underlying text index and through response to suggestions from the community. The web application is available at https://www.kinderminer.org.

## Introduction

Many important scientific discoveries are subject to lengthy processes of trial and error. Because the experimental search spaces are so large, intelligent prioritization of research directions is essential for reaching novel discoveries quickly, and this requires both extensive breadth and depth of domain expertise. However, exponential growth in the scientific literature ^1, 2^ presents a major challenge to remaining conversant with recent knowledge in any domain.

To facilitate rapid prioritization of experimental search, we thus present the first public web application implementation of the KinderMiner algorithm ^3^, built upon a text index of abstracts from PubMed ^4^. The KinderMiner algorithm is a simple text mining algorithm based on co-occurrence counting within a corpus of documents. It addresses the prioritization problem by filtering and ranking a list of target terms (e.g. transcription factors or drugs) by their association with a key phrase (e.g. “embryonic stem cell” or “hypoglycemia”). The output list provides researchers with an informed starting point for understanding the state of literature in their domain, and for prioritizing potential research directions, thereby accelerating the discovery process. While other tools provide similar functionality, we find that KinderMiner’s string matching approach is more flexible and performs comparably or better than existing state-of-the-art tools.

Our web application implementation of KinderMiner improves on the original published algorithm in multiple ways. First, we have constructed our own local biomedical literature index backing the web application. This obviates the need for researchers to produce their own corpus of documents in order to use KinderMiner. Furthermore, providing a local text index speeds up query times, owing to the fact that we no longer need to send repeated queries to a remote web service over the internet. The local index also gives us complete control over data processing, allowing for greater extensibility. Second, providing a graphical user interface increases accessibility over a command-line tool, allowing non-technical users to get results without the bottleneck of relying on computational assistance. The interactive filtering tool also makes it easier for users to visually analyze their results rather than simply picking an arbitrary threshold. Finally, we intend to continually improve the tool by updating the text index quarterly, by adding enhancements, and by acting on feedback from the community. In summary, our application is fast, easy to use, and provides the first publicly available implementation of KinderMiner for all to freely use and to help improve through their feedback.

## Methods

As stated, our web application provides an off-the-shelf implementation of the KinderMiner algorithm built on a provided text corpus derived from PubMed. Here we first briefly describe the KinderMiner algorithm, implementation details of our web application, explain the user interface, and compare our web application results to other state-of-the-art tools on a cell reprogramming task.

### KinderMiner algorithm

**Figure 1.**
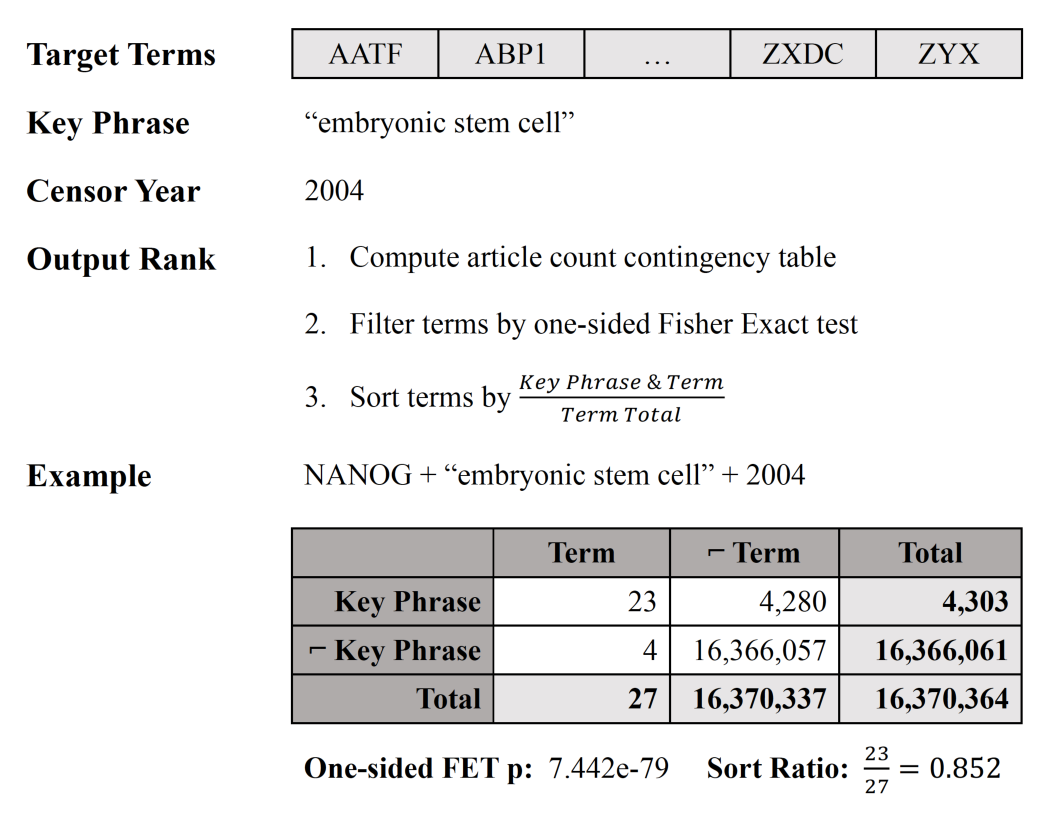
A diagrammatic example of KinderMiner for the key phrase “embryonic stem cell” and target term “NANOG.”

Given a list of target terms and a key phrase of interest, KinderMiner filters and ranks the target terms by their association with the key phrase. It does this via simple string matching and co-occurrence counting within a given document corpus. For every target term in the given list, KinderMiner uses exact token matching to count 1) the number of documents in which the target term occurs, 2) the number of documents in which the key phrase occurs, and 3) the number of documents in which the target term and the key phrase both occur. With these counts, KinderMiner constructs a contingency table of document-level co-occurrence for every target term. KinderMiner then performs a one-sided Fisher’s exact test on every contingency table, and filters out terms that do not meet a specified threshold of co-occurrence significance. Finally, KinderMiner ranks the remaining terms by the ratio of documents containing both the term and key phrase, over the total of documents containing the term, thereby giving a proportion of term association with the key phrase. Figure 1 shows a visual representation of the algorithm steps with an example for a single target term. In the web application, the filtration step is controllable with the interactive analysis tool.

### Implementation

The original KinderMiner publication used Europe PubMed Central ^5^ as the article corpus, but dependency on a remote third-party corpus would be slower and harder to maintain for our web application. Instead, we constructed a local text index from the National Library of Medicine’s “Annual Baseline” Dataset ^4^, containing roughly 30 million abstracts, and updated quarterly by supplementing files from the “Daily Update Files” Dataset. We download all data in XML format. For every PubmedArticle element in the XML, we extract the contents of the PubDate and AbstractText fields. We process the PubDate field into a publication year based on the documentation guidelines and do no further processing on the AbstractText field. We then convert these fields into a JSON format for ingestion by Elasticsearch. Finally, we ingest the converted JSON records into an Elasticsearch index (version 2.4.6). Note that our corpus contains the entirety of the released PubMed citation records, which includes publication records from as far back as the 18th century all the way to the time of ingestion. The results presented here are based on the index built from an ingest of PubMed in June of 2020. The dataset contains 31,030,308 citation records, and we indexed the abstract text with Elasticsearch using the standard analyzer, which applies a grammar-based tokenizer and lowercase filter to the text.

Our web application implements the KinderMiner algorithm built on this provided text index from PubMed. The web application is built with the Flask framework (version 1.0.2) using Python (version 3.7.2), and we use MariaDB (version 5.5.65) for the web application database. When a request is submitted through the application, it is added to the database on a first-come first-serve basis for analysis. Our analysis daemon then uses the Elasticsearch Query Domain Specific Language to construct each of the queries in JSON and stores the counts back in the database for user consumption. Once a request is complete, the results are viewable, filterable, and downloadable.

### Operation

**Figure 2.**
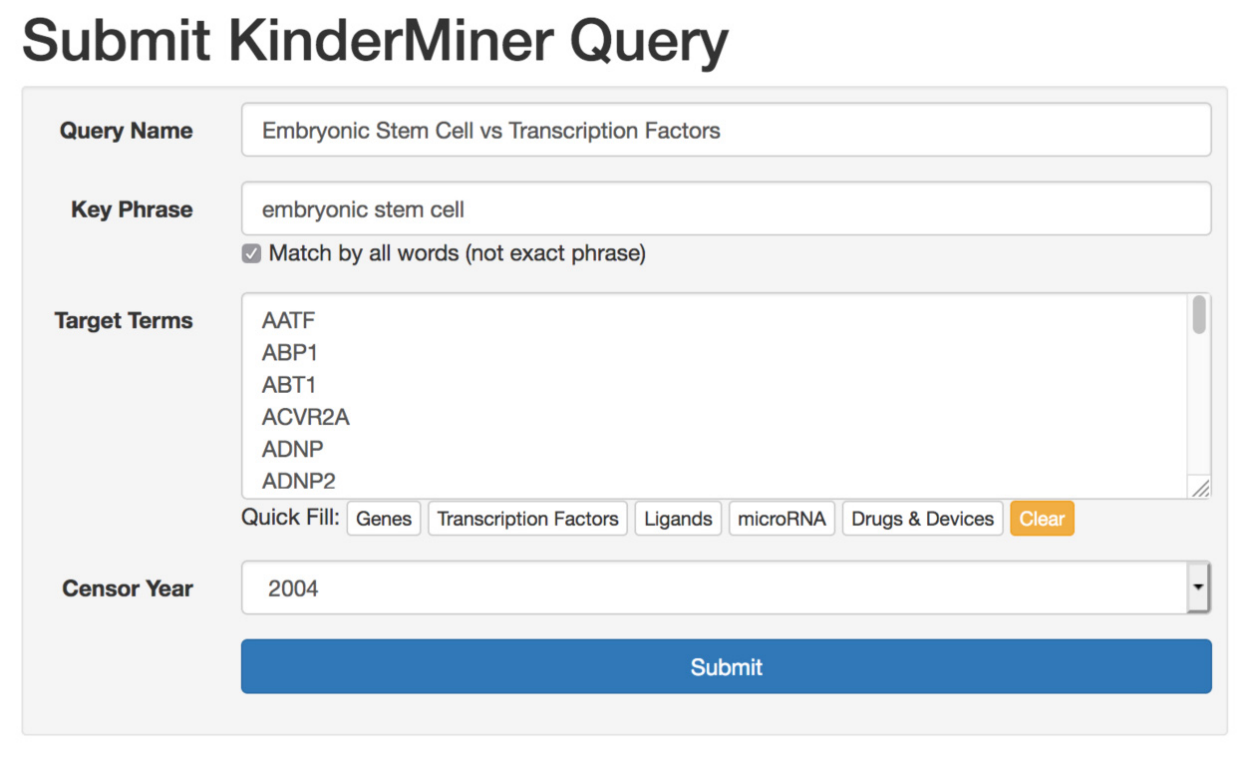
Users enter a search for a particular key phrase and list of target terms.

First, users have the option of either creating an account with their email address or using the application as a guest. With an account, users have indefinite access to all of their previously submitted queries and results. Guests have access to all of the same tools and functionality, except that their query history is limited to their current browser session. The two pages where users will spend most of their time are the query submission page, and the results table for each query. The query submission page (see Figure 2) allows users to submit a single query for a list of target terms and key phrase. On this page, users can name the query for future reference, enter their key phrase, list of target terms, and have the option of selecting an article censor year. The article censor year limits the text search to articles published from the beginning of the text index (18th century) through the end of the specified year, allowing users to see what results may have looked like in years prior. For convenience, we provide quick fill target term lists for genes, transcription factors, ligands, microRNA, and drugs and devices. After submission, queries enter the processing queue. Upon completion, typically within minutes, logged in users receive an email notification.

**Figure 3.**
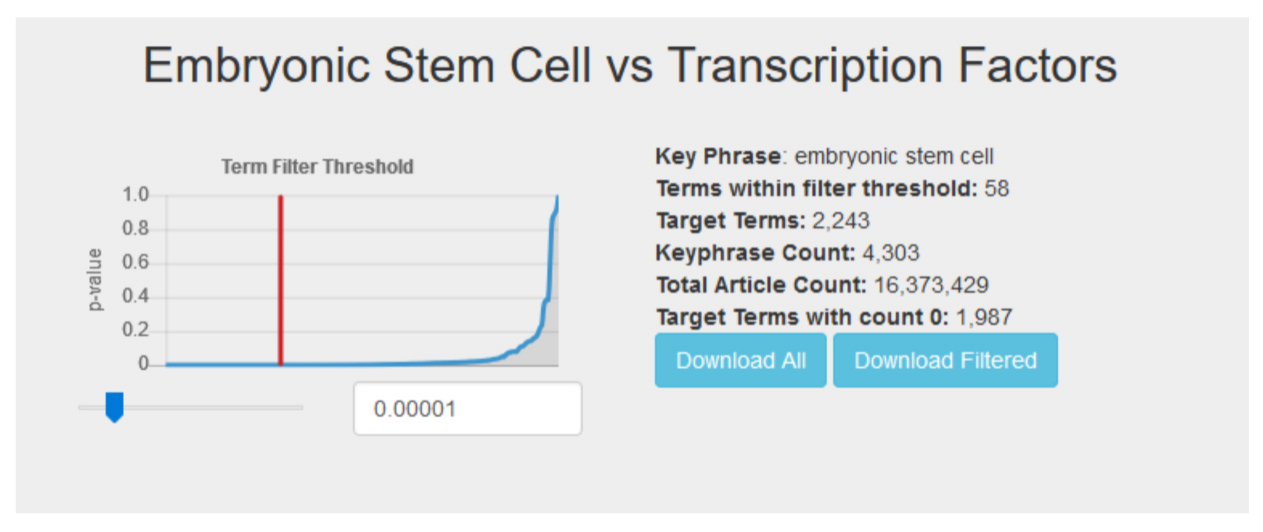
Users can dynamically filter the results for a query using the p-value slider.

When viewing the results table for a particular query (see Figure 3), users are presented with a dynamic list and a p-value threshold slider. The threshold slider controls the Fisher’s exact test p-value by which target terms are filtered, and defaults to a value of 1 × 10 ^−5^. Moving the slider or entering a value in the threshold box automatically updates the content of the displayed term list. A graph shows a curve representing the sorted list of all target term p-values and the current selected cutoff, giving users a visual representation of their filter. With this, users can investigate their top hits further as they see fit. Finally, users also have the option of downloading the entire list of target term counts, or the current filtered list based on their selected threshold.

## Results

Given that the corpus used for our web application is different from the original KinderMiner publication, we need to validate that our new index produces results of similar quality. To do this, we query the same cell reprogramming tasks from the KinderMiner algorithm publication, using the same key phrases, target lists, and censor years. Specifically, we run queries to discover and rank important transcription factors for creating induced pluripotent stem cells (iPS cells), cardiomyocytes, and hepatocytes. For each of the queries, we use the same list of 2,243 transcription factors from the original publication (available as a quick-fill option in the application) and search against the key phrases “embryonic stem cell”, “cardiomyocyte”, and “hepatocyte” respectively. To validate findings for each, we compare the top hits with relevant factors found by the earliest landmark papers for each discovery. Furthermore, we censor each query to only include articles from the earliest publications in our text index (18th century) through December 31 of the year two years prior to the landmark publications (e.g. for the iPS discovery, which was first published in 2006, we include articles through December 31, 2004). Thus, positive findings demonstrate early discovery of the landmark findings and KinderMiner’s potential for prioritizing and expediting the discovery process. For the iPS cell discovery, we censor to articles published through December 31, 2004, and the relevant transcription factors we consider are KLF4, LIN28, MYC, NANOG, POU5F1, and SOX2 ^6– 8^, though we do also note that POU5F1 and SOX2 constitute a sufficient subset for iPS reprogramming ^9^. For cardiomyocytes, we censor to articles published through December 31, 2008, and consider GATA4, HAND2, MEF2C, NKX2-5, and TBX5 ^10, 11^. For hepatocytes, we censor to articles published through December 31, 2009, and consider CEBPB, FOXA3, FOXA2, GATA4, HNF1A, HNF4A, and MYC ^12, 13^. We use a term filter p-value threshold of 1 × 10 ^−5 ^for all of them. In every case, our KinderMiner web application recovers the same positive hits in the top 20 as in the original publication.

However, this initial evaluation does not necessarily confirm that KinderMiner performs any better than other state-of-the-art tools. We thus compare our cell reprogramming results from KinderMiner with those from other similar text mining tools. While there have been many algorithms proposed around the concept of co-occurrence counting, we found only three tools comparable to KinderMiner available: FACTA+ ^14^, Polysearch2 ^15^, and BEST ^16^. There are other similar sounding tools like DeepLife ^17^ and Life-iNet ^18^, but DeepLife serves more as a general biomedical web search than a term ranking tool and Life-iNet does not appear to have any code or application available for use. All three of FACTA+, Polysearch2, and BEST allow the user to rank a list of biomedical entities (analogous to the KinderMiner target terms) by their association with a query entity (analogous to the KinderMiner key phrase), and all allow general text entry for the query entity. Unlike KinderMiner, however, they all perform some form of biomedical entity labeling and indexing for their entity lists. While this approach has advantages, it also limits user queries to the predefined vocabularies of entities that are provided by each tool. KinderMiner is more flexible as it allows users to rank and filter a list of any text terms they like against any text key phrase that they like.

Perhaps the closest comparison tool to KinderMiner is BEST, as it provides a “Transcription Factor” option for one of its predefined entity lists. It also provides the option to censor its corpus search by year, giving us the greatest ability to compare with KinderMiner’s censored results. FACTA+ and Polysearch2 do not have predefined transcription factor lists, but do have “Gene/Protein” and “Genes/Proteins” options respectively. We use these lists as the closest approximation. FACTA+ and Polysearch2 also do not have an option to censor the corpus by year, so they have the advantage of many more years of text as compared to KinderMiner and BEST. For all tools, we use the same three key phrases (“embryonic stem cell”, “cardiomyocyte”, and “hepatocyte”) as query entities. We performed all searches for comparison on February 3, 2020.

Table 1, Table 2, and Table 3 show the top 20 transcription factors determined by each method on these three cell reprogramming tasks. Important transcription factors that appear in the top 20 hits for each method and cell type are highlighted in blue, with duplicate hits highlighted in orange (FACTA+ only). Recall@20 is shown in the bottom row of each table.

**Table 1.** iPS cell transcription factor search. Landmark factors are highlighted in blue (duplicates in orange) and the bottom row shows Recall@20. All methods find a sufficient set of factors (POU5F1 and SOX2). Note that KinderMiner and BEST have been censored to articles published through 2004, whereas the other methods have no such censoring, giving them the advantage of access to the landmark papers and more.

KM-2004	BEST-2004	FACTA+	Polysearch2
NANOG	POU5F1	Oct4 _[POU5F1]_	ESCS
UTF1	LBX1	OCT4	OCT3 _[POU5F1]_ Homeo box transcription factor nanog ANOP-3 _[SOX2]_
POU5F1	TP53	Nanog	
TCF7	TBX1	histone	
FOXD3	GATA1	insulin	DAZ homolog
DNMT3L	FOS	SOX2	Bladder cancer related protein XHL
SOX2	MYC	alkaline phosphatase	Acetyl-CoA carboxylase biotin holoenzyme synthetase
PITX3	STAT3	NANOG	BMP-2B
MYF6	RUNX1	collagen	JARID-2
HIF1A	JUN	p53	FOXD-3
SOX1	HOXB4	nestin	E2A/HLF fusion gene
PDX1	HIF1A	CD34	LIN-41
PAX4	MSC	cytokine	Epithelial zinc finger protein EZF _[KLF4]_
HOXB3	PAX3	leukemia inhibitory factor	APRF
HMGA1	MYF5	osteogenic	MIRN410
LMO2	NEUROD1	catenin	HRIHFB2060
OLIG2	SOX2	gut	ERG associated protein with SET domain
DNMT1	PDX1	erythroid	DMTase
RUNX1	SPI1	c-Myc	BIG-3
HOXB4	SP1	Leukemia inhibitory factor	ER71
**50% (3/6)**	**50% (3/6)**	**67% (4/6)**	**67% (4/6)**

**Table 2.** Cardiomyocyte transcription factor search. Landmark factors are highlighted in blue and the bottom row shows Recall@20. Note that KinderMiner and BEST have been censored to articles published through 2008, whereas the other methods have no such censoring, giving them the advantage of access to the landmark papers and more.

KM-2008	BEST-2008	FACTA+	Polysearch2
GATA4	HLHS2	caspase-3	Adenovirus E4 gene transcription factor 60 kD subunit
NKX2-5	NFKB1	collagen	Apopain
TBX18	AR	angiotensin II	FNDC-5
HDAC9	JUN	Bcl-2	ADCAD-1
TBX20	MSC	ATP	BAG family molecular chaperone regulator 3
NFATC4	TLX2	insulin	Cytoplasmic nuclear factor of activated T-cells 3
GATA5	GATA4	p38	APRF
TBX5	TP53	Ang II	GGF-2
ISL1	STAT3	sarcomeric	GATA binding factor 4
HAND2	PPARA	cardiac muscle	FK506 binding protein 12 rapamycin complex assoc. protein 1
MEF2C	FOS	cytokine	T box 20
NFATC3	NR3C2	natriuretic peptide	5’-AMP-activated protein kinase catalytic subunit alpha-1
HDAC5	HIF1A	ERK1	KKLF
FOXO3A	IRF6	myosin heavy chain	T box 5
GATA6	MEF2A	lactate dehydrogenase	Antigen NY-CO-9
MEF2A	FOSB	endoplasmic reticulum	HMOX-1
ILK	SRF	atrial natriuretic peptide	CASZ-1
SRF	POU5F1	MAPK	AMPH-2
STAT3	TBX5	ATPase	DMDL
MSC	PPARG	tumor necrosis factor	NAD-dependent deacetylase sirtuin
**100% (5/5)**	**40% (2/5)**	**0% (0/5)**	**40% (2/5)**

**Table 3.** Hepatocyte transcription factor search. Landmark factors are highlighted in blue and the bottom row shows Recall@20. Note that KinderMiner and BEST have been censored to articles published through 2009, whereas the other methods have no such censoring, giving them the advantage of access to the landmark papers and more.

KM-2009	BEST-2009	FACTA+	Polysearch2
HNF4A	NFKB1	hepatocyte growth factor	Acetyl-CoA carboxylase biotin holoenzyme synthetase
HNF1A	IRF6	albumin	HNF-4
HNF1B	TP53	insulin	ABC16
TCF2	HNF4A	cytokine	F TCF
TCF1	MYC	c-Met	ABC30
FOXA3	JUN	collagen	EGF receptor
NR1I3	PPARA	HGF	5’-AMP-activated protein kinase catalytic subunit alpha-1
NR0B2	ESR1	epidermal growth factor	AQP-7
FOXA2	HNF1A	VEGF	APRF
NR1I2	STAT3	cytochrome P450	FABP-1
NR1H4	NR3C1	alanine aminotransferase	ACT2
IPF1	FOSB	tumor necrosis factor	HAMP
FOXA1	NR1I2	scatter factor	Apopain
FOXF1	AHR	endoplasmic reticulum	HGF receptor
PBX2	FOS	Met	C8FW
NEUROD1	PPARG	MET	NR1C1
PROX1	MBD2	aspartate aminotransferase	CPE-1
ALF	ONECUT1	ATP	NTCP
PAX4	HNF1B	IL-6	KLHL-1
FOXO1A	FKHL16	caspase-3	SREBF-1
**57% (4/7)**	**43% (3/7)**	**0% (0/7)**	**14% (1/7)**

## Use cases

Of course, KinderMiner is designed to be general enough to work for other biomedical applications. In fact, it has already been used as part of several other published applications. In one, KinderMiner was used to validate phenotypes found to be associated with FMR1 premutation as part of electronic health record (EHR) analysis ^19^. In that case, KinderMiner helped provide evidence that FMR1 premutation carriers experience a clinical profile different from that of a control population. In another application, KinderMiner was used to assess novelty of lab tests as predictors for certain diseases ^20^. In that work, EHR analysis revealed that common lab tests are sometimes predictive of diagnoses for which they would not typically be used. KinderMiner was used to validate the novelty of those findings by using the opposite-handed statistical test and an inverse ranking function. KinderMiner has also been used to identify protein-protein interactions ^21^, outperforming Polysearch2 in that work as well. Finally, the original KinderMiner publication also demonstrated its use to identify potential drug repositioning candidates for diabetes ^3^, finding several relevant hits and providing comparable results to a more sophisticated computational approach.

## Discussion

From Table 1, we note that all methods perform comparably on the iPS cell reprogramming task, and all do in fact find a sufficient set of reprogramming factors ^9^ (POU5F1 and SOX2) in the top hits. Recall again, however, that FACTA+ and Polysearch2 have access to literature available years after the landmark discoveries were made, whereas KinderMiner and BEST have both been censored to articles published through 2004 (two years prior to discovery). KinderMiner also finds all five relevant factors for cardiomyocytes in the top 11 hits of Table 2, and finds most factors for hepatocyte reprogramming in the top nine hits of Table 3, outperforming the comparison methods by recall for both cardiomyocyte and hepatocyte reprogramming. Furthermore, KinderMiner is not limited to predefined vocabularies like all of the comparison methods. The string matching and counting approach used by KinderMiner is both simpler and more flexible while performing comparably if not better than the predefined vocabulary approach. In general, approaches like KinderMiner’s tend to achieve high recall without requiring annotated training data ^22^.

Even just these three results show how valuable KinderMiner can be in a research work flow. The resulting list provided by KinderMiner not only provides researchers with suggested reading, but also allows them to prioritize their targets for experimentation. Consider the discovery of how to make iPS cells before it was known. If one assumes a priori that 2–3 transcription factors are needed, then the task quickly becomes unmanageable without some prioritization of the roughly 2,000 human transcription factors ( $\left(\begin{array}{c} 2,000\\ 2 \end{array}\right)=2.0\times 10^{6}$ and $\left(\begin{array}{c} 2,000\\ 3 \end{array}\right)=1.3\times 10^{9}$ ). If a researcher wants to know if NANOG is associated with pluripotency, they can use a search engine to find and read specific articles about that single connection. That, however, is only one finding, and the researcher has to know what connection they are looking for (NANOG and pluripotency) beforehand. If a researcher instead wants to know which of all roughly 2,000 transcription factors are most likely associated with pluripotency according to the current state of the literature, the required reading would be infeasible. Furthermore, after extensive reading, the researcher still needs to synthesize that knowledge into an ordered set of the most promising leads to try as reprogramming factors to make iPS cells. This is exactly the type of situation where KinderMiner shines. In a matter of seconds to minutes, that same researcher can get an ordered list of promising leads to help them prioritize their reading or experimentation.

## Limitations and future work

While KinderMiner performs well empirically, it is not without limitations. One potential shortcoming is the lack of negation handling. Because KinderMiner only looks for document level co-occurrence, it cannot distinguish between a positive or negative association. For example, if many articles contain phrases like “Gene A is not associated with tissue B,” KinderMiner will still likely pick up on this relation between gene A and tissue B and produce it as a significant hit. We are currently exploring options for addressing negation. Nevertheless, even with this lack of negation handling, KinderMiner performs well on a variety of tasks.

Another potential shortcoming of KinderMiner is that, in some cases, the exact matching approach requires more curation from the user. Exact text matches are immediately useful when the list of target terms is something like genes, where well-defined lists are available and where it may be important to distinguish between similar names like TWIST1 and TWIST2, but it becomes more challenging when the target term list is more complicated. For example, a target term list of ICD9 codes would be more challenging. ICD diagnosis descriptions are often very specific or contain tokens that would not typically appear in the literature, thus requiring curation if they are to be used for a target term list. For example, “Malignant neoplasm of breast (female); unspecified site” is unlikely to occur as an exact string or set of tokens in the literature, so this term would require manual modification (e.g. to “malignant breast cancer”) before use with KinderMiner. This minor difficulty is effectively a tradeoff made in exchange for the flexibility of being able to use any list of target terms as text.

One possible way to alleviate some of limitations of exact string matching is to build in a synonym matcher. KinderMiner does not have this feature at this time. Thus, a match to POU5F1, for example, will not also include matches to OCT4. Similarly, KinderMiner does not currently perform any stemming, which means that tokens like “pluripotent” and “pluripotency” are not counted identically. KinderMiner still performs well without either synonym matching or stemming, and while they may increase true hits, they may also increase false hits. Nevertheless, synonym matching and stemming are areas of future work that we are actively working on and evaluating.

Further areas of interest include using named entity recognition to help disambiguate tokens like the gene “WAS” from the verb, features to filter the corpus content by more than just publication year, and Bayesian methods to modulate term ranks. Of course, this is not an exhaustive list of possible improvements. There are numerous research directions we may investigate and incorporate into the tool as they prove useful.

Regarding KinderMiner’s speed, the primary factor that determines the time to complete a request is the length of the target term list. Based on our own tests with target term lists ranging from thousands to tens of thousands in length, requests currently take roughly 12 milliseconds per target term. Thus, a request on a list of roughly 2,000 transcription factors works out to around 24 seconds, or around 4 minutes for a request on all roughly 20,000 human genes. Of course, heavy traffic on the web application could also affect response time as requests are queued on a first-come first-serve basis. We do not anticipate an issue in the short term, but we are actively investigating queueing and batch querying options to improve speed and user experience even further.

Finally, while we consider the KinderMiner web application to be a living tool that will improve and change over time, we want to be able to provide users with reproducible results. To address this, we eventually intend to allow users to select from a backlog of text indices going back one or two years.

## Conclusions

We present the first publicly available implementation of the KinderMiner algorithm. It includes a user-friendly interface and is built on top of a fast and local index of PubMed abstracts. We demonstrate the utility of the KinderMiner web application on the task of identifying transcription factors likely to be useful to reprogram cells to a particular state, but the tool is general and can be used to help prioritize any biomedical experiment or address any biomedical question of interest to the user.

Our example results suggest that, even though KinderMiner is simple and derives its results from correlations already present in the literature, it can synthesize those correlations into a coherent single discovery not yet commonly known. We plan to continue to improve the KinderMiner web application with quarterly updates, by addressing limitations, improving the interface, and by responding to suggestions from the community.

## Data availability

The PubMed abstract corpus we use is available for download as the National Library of Medicine’s “Annual Baseline” Dataset ^4^.

## Software availability

The KinderMiner web application is freely available for use by everyone at https://www.kinderminer.org. 

Code to download, process, and index PubMed abstracts is available at https://github.com/iross/km_indexer.

Archived code as at time of publication: https://doi.org/10.5281/zenodo.3948498 ^23^.

License: MIT

Code for the web application itself is available at https://github.com/stewart-lab/kinderminer_webapp.

Archived code as at time of publication: https://doi.org/10.5281/zenodo.3947008 ^24^.

License: MIT
