# Supplementary material for: KinderMiner Web: a simple web tool for ranking pairwise associations in biomedical applications
Source: F1000Res. 2021 Dec 20;9:832. Originally published 2020 Jul 30. [Version 2] doi: 10.12688/f1000research.25523.2 (PMC8756297; doi:10.12688/f1000research.25523.2)
Supplement: Peer review history

# Peer review: Reviewer response for version 2

**DOI:** 10.5256/f1000research.81433.r115918

**Published:** 2022-01-12

**Authors:** Qingyu Chen

**Affiliations:** 1 National Center for Biotechnology Information (NCBI), National Library of Medicine (NLM), National Institutes of Health (NIH), Bethesda, MD, USA

Thanks to the authors for their efforts in revising the paper. My main comments have been addressed.

Are the conclusions about the tool and its performance adequately supported by the findings presented in the article?

Partly

Is the rationale for developing the new software tool clearly explained?

Yes

Is the description of the software tool technically sound?

Partly

Are sufficient details of the code, methods and analysis (if applicable) provided to allow replication of the software development and its use by others?

Yes

Is sufficient information provided to allow interpretation of the expected output datasets and any results generated using the tool?

Yes

Reviewer Expertise:

I assess mainly based on the computational side. The impacts on the biomedical knowledge side also need to be assessed.

I confirm that I have read this submission and believe that I have an appropriate level of expertise to confirm that it is of an acceptable scientific standard.

# Peer review: Reviewer response for version 2

**DOI:** 10.5256/f1000research.81433.r115917

**Published:** 2021-12-21

**Authors:** Sylvester O. Orimaye

**Affiliations:** 1 College of Public Health, East Tennessee State University, Johnson City, TN, USA

I have no further comments to make.

Are the conclusions about the tool and its performance adequately supported by the findings presented in the article?

Yes

Is the rationale for developing the new software tool clearly explained?

Yes

Is the description of the software tool technically sound?

Yes

Are sufficient details of the code, methods and analysis (if applicable) provided to allow replication of the software development and its use by others?

Yes

Is sufficient information provided to allow interpretation of the expected output datasets and any results generated using the tool?

Yes

Reviewer Expertise:

Natural Language Processing, Machine Learning, Literature-Based Discovery, Computational Linguistics, Bioinformatics, Biostatistics, Public Health.

I confirm that I have read this submission and believe that I have an appropriate level of expertise to confirm that it is of an acceptable scientific standard.

# Peer review: Reviewer response for version 1

**DOI:** 10.5256/f1000research.28167.r83813

**Published:** 2021-05-12

**Authors:** Sylvester O. Orimaye

**Affiliations:** 1 College of Public Health, East Tennessee State University, Johnson City, TN, USA

The authors presented KinderMiner web interface for ranking biomedical experiments. The KinderMiner algorithm was previously published and can be used for other Biomedical knowledge search tasks such as identifying transcription factors for reprogramming cells.

 While there are a series of limitations around the current version of the algorithm and the web interface, as stated by the authors, the web interface could benefit users in terms of user-friendliness and efficiency (search response time). In particular, the lack of latent semantic processing capability could significantly limit the results of the algorithm. It cannot be over-emphasized that semantically related concepts found in many ontologies have increasingly become an essential part of search engines. The authors should seriously consider this concept in their future versions.

 The authors could also explain the rationale behind setting the Fisher's exact threshold to 0.00005. How was the point determined? What would be the difference (in terms of recall or p@20) between the results if a different threshold was to be used? Would it be statistically significant?

 I found minor typos such as " we need to validate..." even though the authors described the experiment in the past. Please check for other typos in the final version of the manuscript.

Are the conclusions about the tool and its performance adequately supported by the findings presented in the article?

Yes

Is the rationale for developing the new software tool clearly explained?

Yes

Is the description of the software tool technically sound?

Yes

Are sufficient details of the code, methods and analysis (if applicable) provided to allow replication of the software development and its use by others?

Yes

Is sufficient information provided to allow interpretation of the expected output datasets and any results generated using the tool?

Yes

Reviewer Expertise:

Natural Language Processing, Machine Learning, Literature-Based Discovery, Computational Linguistics, Bioinformatics, Biostatistics, Public Health.

I confirm that I have read this submission and believe that I have an appropriate level of expertise to confirm that it is of an acceptable scientific standard.

# Response

**Published:** 2021-12-13

**Authors:** Finn Kuusisto

**Affiliations:** Morgridge Institute for Research, USA

*We greatly appreciate the Dr. Orimaye's patience, time, and effort in providing useful feedback and corrections.*

 > The authors could also explain the rationale behind setting the Fisher's exact threshold to 0.00005.

*We used 0.00001 for the Fisher’s Exact Test threshold. In this case we simply used the same search parameters as those that we used in the original KinderMiner algorithm publication. That said, we did not have a rigorous statistical justification for the threshold in the original paper. Instead, we chose the 1e-5 threshold, as it frequently resulted in final ranked lists in the range of 50 to 100 hits, which was a reasonable size when presenting results to collaborators working at the bench. We stuck with that choice for our own internal use of the algorithm and for comparison to the original here, but we provide the interactive slider in the webapp for flexibility and exploration of resultant list sizes. We have elaborated on this choice in the paper.*

 > It cannot be over-emphasized that semantically related concepts found in many ontologies have increasingly become an essential part of search engines.

*Dr. Chen and Dr. Orimaye have both commented on the limitations of using exact string matching. While we agree that exact string matching has potential limitations, we argue that exact string matching has advantages as well. Primarily, exact string matching allows for rapid exploration of the literature versus ontologies or semantic matching methods. Building ontologies or semantic models for any category of biomedical entity requires a great deal of time and effort, whereas exact matching allows for the quick creation of custom lists of entities of interest, which we found to be a common desire among our colleagues at the bench. Instead of looking for a tool that provides a predefined entity set that seems closest to their interests, they can simply create a list on the fly and run a search. Furthermore, as new terms arise (e.g., genes or drugs), the ontologies or semantic models need to be reworked/retrained rather than simply adding a string to the end of a list. The trade-off is that some entities may require manual curation and a possible sacrifice in recall in some cases. Further, semantic matching approaches, or even simple synonym matching as we described with the embryonic transcription factor above, have the potential to introduce errors of their own when the simple exact match works as is. That said, we understand and appreciate the concern for our approach, have attempted to clarify this in the limitations section, and have elaborated on how we are considering more advanced matching for future versions.*

 > I found minor typos such as " we need to validate..." even though the authors described the experiment in the past. Please check for other typos in the final version of the manuscript.

*We have corrected that typo and have checked the manuscript for other typos.*

*We greatly appreciate Dr. Orimaye's patience, time, and feedback. We intend for this software tool to give more researchers access to a simple algorithm that has helped us to prioritize some of our own research over the years. Overall, despite limitations, we find that the empirical results speak for themselves and hope that others will find it useful too.*

# Peer review: Reviewer response for version 1

**DOI:** 10.5256/f1000research.28167.r82000

**Published:** 2021-04-19

**Authors:** Qingyu Chen

**Affiliations:** 1 National Center for Biotechnology Information (NCBI), National Library of Medicine (NLM), National Institutes of Health (NIH), Bethesda, MD, USA

This paper describes a tool for efficient discovery on potential associations of terms in biomedical literature. Given a list of terms of interest and a query phrase, it matches and ranks the documents at the abstract level. It also provides a case study to demonstrate its usage. The tools are also publicly available. I have a few comments on the evaluation, limitation, and functionality summarized below.

 My primary comment is the precision and recall of the term matching part should be quantified. Given only a simple string matching method is used, it will potentially miss identifying the same entities using different expressions or wrongly identify the entities using exact terms but represent differently (for example, some genes share the same names with chemicals). Importantly, this is the first step of the algorithm; errors would propagate to the later stages. It is therefore critical to provide a detailed evaluation on this part. In addition, does the indexing part incorporate synonyms? The descriptions are not very clear. 

 Other comments are relatively minor. A primary comment is the limitation should also specify the application is limited to the abstract level only. Also, in terms of the function, please considering providing an API so that potential users can query the associations systematically.

Are the conclusions about the tool and its performance adequately supported by the findings presented in the article?

Partly

Is the rationale for developing the new software tool clearly explained?

Yes

Is the description of the software tool technically sound?

Partly

Are sufficient details of the code, methods and analysis (if applicable) provided to allow replication of the software development and its use by others?

Yes

Is sufficient information provided to allow interpretation of the expected output datasets and any results generated using the tool?

Yes

Reviewer Expertise:

I assess mainly based on the computational side. The impacts on the biomedical knowledge side also need to be assessed.

I confirm that I have read this submission and believe that I have an appropriate level of expertise to confirm that it is of an acceptable scientific standard, however I have significant reservations, as outlined above.

# Response

**Published:** 2021-12-13

**Authors:** Finn Kuusisto

**Affiliations:** Morgridge Institute for Research, USA

*We greatly appreciate Dr. Chen's patience, time, and effort in providing useful feedback and corrections.*

 > A primary comment is the limitation should also specify the application is limited to the abstract level only.

*We did not emphasize the fact that our search is only at the abstract level in the limitations section. This was simply an oversight, and we have now added it to the limitations section.*

 > Please consider providing an API so that potential users can query the associations systematically.

*This is certainly a feature we have considered, and we intend to update the software with new backend data and features according to user demand. If it becomes a common request, we will absolutely implement it.*

 > In addition, does the indexing part incorporate synonyms?

*We do not perform any synonym matching as implemented but have explored it as an option and do ultimately intend to incorporate some version of it in the future. One major issue that we have found with synonym matching though is that, while it has the potential to increase recall, it can also substantially decrease precision. For example, one synonym of the gene name POU5F1 is OCT3, but OCT3 is also a synonym of SLC22A3, and this is not a particularly unique case even in the simple domain of gene names. Similar situations also frequently occur with acronyms, such as DR for Diabetic Retinopathy.*

*To elaborate further, in our exploration of synonym matching we ran the “embryonic stem cell” example with and without synonyms. While POU5F1, NANOG, and SOX2 show up in the top 7 hits without synonyms, the search with synonyms didn’t even include SOX2 in the top 20 and NANOG was pushed to hit 16. This is due at least in part to several hits getting inflated counts due to synonym collisions with other words and each other. SPI1, TAF1, PAX6, and NR4A2 show up as hits number 3, 4, 5, and 7 respectively when we ran the search with synonyms. This is likely because both SPI1 and TAF1 have the synonym “OF,” PAX6 has a synonym “AN,” and NR4A2 has a synonym “NOT.” It appears then that synonym matching alone could very possibly lead to a substantial tradeoff in precision. We have clarified that KinderMiner does not perform synonym matching and increased emphasis of this point in the limitations section.*

*Given that this a software paper rather than a full research paper, we chose to share this tool with the simpler approach of exact string matching without synonyms because of its flexibility and because it has been demonstrated to work well for us under most circumstances.*

 > My primary comment is the precision and recall of the term matching part should be quantified. Given only a simple string matching method is used, it will potentially miss identifying the same entities using different expressions or wrongly identify the entities using exact terms but represent differently (for example, some genes share the same names with chemicals). Importantly, this is the first step of the algorithm; errors would propagate to the later stages.

*Dr. Chen and Dr. Orimaye have both commented on the limitations of using exact string matching. While we agree that exact string matching has potential limitations, we argue that exact string matching has advantages as well. Primarily, exact string matching allows for rapid exploration of the literature versus ontologies or semantic matching methods. Building ontologies or semantic models for any category of biomedical entity requires a great deal of time and effort, whereas exact matching allows for the quick creation of custom lists of entities of interest, which we found to be a common desire among our colleagues at the bench. Instead of looking for a tool that provides a predefined entity set that seems closest to their interests, they can simply create a list on the fly and run a search. Furthermore, as new terms arise (e.g., genes or drugs), the ontologies or semantic models need to be reworked/retrained rather than simply adding a string to the end of a list. The trade-off is that some entities may require manual curation and a possible sacrifice in recall in some cases. Further, semantic matching approaches, or even simple synonym matching as we described with the embryonic transcription factor above, have the potential to introduce errors of their own when the simple exact match works as is. That said, we understand and appreciate the concern for our approach, have attempted to clarify this in the limitations section, and have elaborated on how we are considering more advanced matching for future versions.*

 > It is therefore critical to provide a detailed evaluation on this part.

*Dr. Chen has requested a detailed evaluation of our choice to use exact string matching versus others. Given that this paper is on a software implementation of a previously published algorithm, and because the flexibility afforded by allowing any list of entities makes an exhaustive evaluation of recall and precision an expansive research undertaking, we feel this request is out of scope for this paper. Even an evaluation of recall for our exact string matching on a single gene, such as HNF1A, would require a gold standard labeling of HNF1A-related abstracts for our entire index. In order to more generally evaluate how our exact matching performs on the entire human gene domain would then require nearly 20000 more distinct gold standard abstract labelings of our index. This in turn would be true of every other domain that our algorithm might encounter, including entity lists like proteins, drugs, diseases, cell types, species names, and so on. Because the output of the algorithm is an ordered subset of entities of interest, we have opted instead to present compelling results on several gold standard lists for important discoveries, rather than focusing on how the algorithm may miss individual entities of interest. Exact string matching provides great flexibility by allowing a user to search any list of entities, but this comes at the risk that some important entities may not surface due to say a particularly unpopular spelling. We think the flexibility is worth the tradeoff and that the promising results we have seen justify the decision.*

*We greatly appreciate the Dr. Chen's patience, time, and feedback. We intend for this software tool to give more researchers access to a simple algorithm that has helped us to prioritize some of our own research over the years. Overall, despite limitations, we find that the empirical results speak for themselves and hope that others will find it useful too.*
